# Characteristics of the cervical spine and cervical cord injuries in older adults with cervical ossification of the posterior longitudinal ligament

**DOI:** 10.1038/s41598-023-29877-2

**Published:** 2023-02-15

**Authors:** Shun Okuwaki, Toru Funayama, Masao Koda, Fumihiko Eto, Akihiro Yamaji, Noriaki Yokogawa, Takeshi Sasagawa, Kei Ando, Hiroaki Nakashima, Naoki Segi, Kota Watanabe, Satoshi Nori, Kazuki Takeda, Takeo Furuya, Atsushi Yunde, Hideaki Nakajima, Tomohiro Yamada, Tomohiko Hasegawa, Yoshinori Terashima, Ryosuke Hirota, Hidenori Suzuki, Yasuaki Imajo, Shota Ikegami, Masashi Uehara, Hitoshi Tonomura, Munehiro Sakata, Ko Hashimoto, Yoshito Onoda, Kenichi Kawaguchi, Yohei Haruta, Nobuyuki Suzuki, Kenji Kato, Hiroshi Uei, Hirokatsu Sawada, Kazuo Nakanishi, Kosuke Misaki, Hidetomi Terai, Koji Tamai, Eiki Shirasawa, Gen Inoue, Kenichiro Kakutani, Yuji Kakiuchi, Katsuhito Kiyasu, Hiroyuki Tominaga, Hiroto Tokumoto, Yoichi Iizuka, Eiji Takasawa, Koji Akeda, Norihiko Takegami, Haruki Funao, Yasushi Oshima, Takashi Kaito, Daisuke Sakai, Toshitaka Yoshii, Tetsuro Ohba, Bungo Otsuki, Shoji Seki, Masashi Miyazaki, Masayuki Ishihara, Seiji Okada, Shiro Imagama, Satoshi Kato

**Affiliations:** 1 Department of Orthopaedic Surgery, Faculty of Medicine, University of Tsukuba, 1-1-1 Tennodai, Tsukuba, Ibaraki 305–8575 Japan; 2 Department of Orthopaedic Surgery, Graduate School of Comprehensive Human Sciences, University of Tsukuba, 1-1-1 Tennodai, Tsukuba, Ibaraki 305–8575 Japan; 3 Department of Orthopaedic Surgery, Ibaraki Seinan Medical Center Hospital, 2190, Sakaimachi, Sashima, Ibaraki 306–0433 Japan; 4 Department of Orthopaedic Surgery, Graduate School of Medical Sciences, Kanazawa University, 13–1 Takara-machi, Kanazawa, Ishikawa 920–8641 Japan; 5 Department of Orthopedics Surgery, Toyama Prefectural Central Hospital, 2-2-78 Nishinagae, Toyama, Toyama 930–8550 Japan; 6 Department of Orthopedic Surgery, Nagoya University, Graduate School of Medicine, Nagoya, 65 Tsurumai-cho, Showa-ku, Nagoya, 466–8550 Japan; 7 Department of Orthopaedic Surgery, Keio University School of Medicine, 35 Shinanomachi, Shinjuku-ku, Tokyo, 160–8582 Japan; 8 Department of Orthopaedic Surgery, Japanese Red Cross Shizuoka Hospital, 8–2 Otemachi, Aoi-ku, Shizuoka, 420–0853 Japan; 9 Department of Orthopaedic Surgery, Graduate School of Medicine, Chiba University, 1-8-1 Inohana, Chuo-ku, Chiba, Chiba 260–8670 Japan; 10 Department of Orthopaedics and Rehabilitation Medicine, Faculty of Medical Sciences, University of Fukui, 23–3 Matsuoka Shimoaizuki, Eiheiji-cho, Yoshida-gun, Fukui, 910–1193 Japan; 11 Department of Orthopaedic Surgery, Hamamatsu University School of Medicine, 1-20-1, Handayama, Higashi-ku, Hamamatsu, Shizuoka 431–3192 Japan; 12 Department of Orthopaedic Surgery, Nagoya Kyoritsu Hospital, 1–172 Hokke, Nakagawa-ku, Nagoya-shi, Aichi 454–0933 Japan; 13 Department of Orthopaedic Surgery, Sapporo Medical University, South 1-West 16–291, Chuo-ku, Sapporo, 060–8543 Japan; 14 Department of Orthopaedic Surgery, Matsuda Orthopedic Memorial Hospital, North 18-East 4–1 Kita-ku, Sapporo, 001–0018 Japan; 15 Department of Orthopaedic Surgery, Yamaguchi University Graduate School of Medicine, 1-1-1 Minami-Kogushi, Ube, Yamaguchi 755–8505 Japan; 16 Department of Orthopaedic Surgery, Shinshu University School of Medicine, 3-1-1 Asahi, Matsumoto, Nagano 390–8621 Japan; 17 Department of Orthopaedics, Graduate School of Medical Science, Kyoto Prefectural University of Medicine, Kawaramachi-Hirokoji, Kamigyo-ku, Kyoto, 602–8566 Japan; 18 Department of Orthopaedics, Saiseikai Shiga Hospital, 2-4-1 Ohashi Ritto, Shiga, 520–3046 Japan; 19 Department of Orthopaedic Surgery, Tohoku University Graduate School of Medicine, 1–1 Seiryo-machi, Aoba-ku, Sendai, Miyagi 980–8574 Japan; 20 Department of Orthopaedic Surgery, Graduate School of Medical Sciences, Kyushu University, 3-1-1 Maidashi Higashi-ku, Fukuoka, 812–8582 Japan; 21 Department of Orthopaedic Surgery, Nagoya City University Graduate School of Medical Sciences, 1 Kawasumi, Mizuho-cho, Mizuho-ku, Nagoya, 467–8601 Japan; 22 Department of Orthopaedic Surgery, Nihon University Hospital, 1–6 Kanda-Surugadai, Chiyoda-ku, Tokyo, 101–8393 Japan; 23 Department of Orthopaedic Surgery, Nihon University School of Medicine, 30–1 Oyaguchi Kami-cho, Itabashi-ku, Tokyo, 173–8610 Japan; 24 Department of Orthopedics, Traumatology and Spine Surgery, Kawasaki Medical School, 577, Matsushima, Kurashiki, Okayama 701–0192 Japan; 25 Department of Orthopaedic Surgery, Osaka Metropolitan University Graduate School of Medicine, 1-4-3 Asahimachi, Abeno-ku, Osaka, Osaka 545–8585 Japan; 26 Department of Orthopaedic Surgery, Kitasato University School of Medicine, 1-15-1, Kitazato, Minami-ku, Sagamihara, Kanagawa 252–0374 Japan; 27 Department of Orthopaedic Surgery, Kobe University Graduate School of Medicine, 7-5-1 Kusunoki-cho, Chuo-ku, Kobe, 650–0017 Japan; 28 Department of Orthopaedic Surgery, Kochi Medical School, Kochi University, Kohasu, Oko-cho, Nankoku, 783–8505 Japan; 29 Department of Orthopaedic Surgery, Graduate School of Medical and Dental Sciences, Kagoshima University, 8-35-1 Sakuragaoka, Kagoshima, 890–8520 Japan; 30 Department of Orthopaedic Surgery, Gunma University, Graduate School of Medicine, 3-39-22 Showa, Maebashi, Gunma 371–8511 Japan; 31 Department of Orthopaedic Surgery, Mie University Graduate School of Medicine, 2–174 Edobashi, Tsu, Mie 514–8507 Japan; 32 Department of Orthopaedic Surgery, School of Medicine, International University of Health and Welfare, 852 Hatakeda, Narita, Chiba 286–0124 Japan; 33 Department of Orthopaedic Surgery, International University of Health and Welfare Narita Hospital, 852 Hatakeda, Narita, Chiba 286–0124 Japan; 34 Department of Orthopaedic Surgery and Spine and Spinal Cord Center, International University of Health and Welfare Mita Hospital, 1-4-3 Mita, Minato-ku, Tokyo, 108–8329 Japan; 35 Department of Orthopaedic Surgery, The University of Tokyo Hospital, 7-3-1 Hongo, Bunkyo-ku, Tokyo, 113–8655 Japan; 36 Department of Orthopaedic Surgery, Osaka University Graduate School of Medicine, 2–2 Yamadaoka, Suita, Osaka 565–0871 Japan; 37 Department of Orthopedics Surgery, Surgical Science, Tokai University School of Medicine, 143 Shimokasuya, Isehara, Kanagawa 259–1193 Japan; 38 Department of Orthopaedic Surgery, Tokyo Medical and Dental University, Yushima 1-5-45, Bunkyo-ku, Tokyo, 113–8519 Japan; 39 Department of Orthopaedic Surgery, University of Yamanashi, 1110 Shimokato, Chuo, Yamanashi 409–3898 Japan; 40 Department of Orthopaedic Surgery, Graduate School of Medicine, Kyoto University, 54 Shogoin-Kawaracho, Sakyo-ku, Kyoto, Kyoto Japan; 41 Department of Orthopaedic Surgery, Faculty of Medicine, University of Toyama, 2630 Sugitani, Toyama, Toyama 930–0194 Japan; 42 Department of Orthopaedic Surgery, Faculty of Medicine, Oita University, 1–1 Idaigaoka, Hasama-machi, Yufu-shi, Oita, 879–5593 Japan; 43 Department of Orthopaedic Surgery, Kansai Medical University Hospital, 2-3-1 Shinmachi, Hirakata, Osaka 573–1191 Japan

**Keywords:** Spine structure, Outcomes research, Epidemiology

## Abstract

Although the incidence of cervical spinal cord injury (CSCI) with ossification of the posterior longitudinal ligament (OPLL) has increased in older adults, its etiology and neurological outcomes remain unknown. We identified OPLL characteristics and determined whether they influence neurological severity and improvement of CSCI in older patients. This multicenter retrospective cohort study identified 1512 patients aged ≥ 65 years diagnosed with CSCI on admission during 2010–2020. We analyzed CSCI etiology in OPLL patients. We performed propensity score-adjusted analyses to compare neurological outcomes between patients with and without OPLL. Cases were matched based on variables influencing neurological prognosis. The primary neurological outcome was rated according to the American Spine Injury Association (ASIA) impairment scale (AIS) and ASIA motor score (AMS). In 332 OPLL patients, the male-to-female ratio was approximately 4:1. Half of all patients displayed low-energy trauma-induced injury and one-third had CSCI without a bony injury. Propensity score matching created 279 pairs. There was no significant difference in the AIS grade and AMS between patients with and without OPLL during hospitalization, 6 months, and 12 months following injury. OPLL patients tended to exhibit worse neurological findings during injury; nevertheless, OPLL was not associated with poor neurological improvement in older CSCI patients.

## Introduction

Cervical spinal cord injury (CSCI) in the older adults is expected to increase with an increase in the aging population^1^, and related falls among the older adults in recent decades, thus posing a serious public health concern^2,3^. CSCI in older adults is related to preexisting canal stenosis owing to the ossification of the posterior longitudinal ligament (OPLL)^4^. OPLL of the cervical spine is an inflammatory process that causes the replacement of the posterior longitudinal ligament by the lamellar bone, thereby resulting in spinal cord compression. Moreover, cervical OPLL develops in individuals in their 30 s and 40 s, and the progression halts in patients over 65 years^5–7^.

The presence of OPLL is a risk factor for CSCI. A recent nationwide cohort study investigating the impact of OPLL on the occurrence, severity, and prognosis of CSCI reported that OPLL is a risk factor for CSCI, and this risk is mitigated by surgical treatment^8^. Another study demonstrated that patients conservatively managed with OPLL displayed a 4.8-fold higher risk for CSCI than an age- and sex-matched population without OPLL^9^. Moreover, the influences of OPLL on CSCI are particularly strong for CSCI without bone injury^4,10^. CSCI without bone injury is increasing, a trend that may be related to OPLL and the aging population worldwide^2,3^. In individuals with cervical OPLL, the onset of CSCI is caused by trauma. In addition, it displays a poor prognosis^11,12^. Cervical myelopathy with OPLL leads to poorer postoperative outcomes and neurological improvement rates with cervical laminoplasty, compared with degenerative cervical myelopathy^13^. However, the epidemiology, severity, and prognosis of traumatic CSCI with OPLL in older adults are unknown.

We retrospectively evaluated a large number of older adults (aged ≥ 65 years) with CSCI and OPLL in the Japanese population. We aimed to identify the patient’s background characteristics and determine their impact on the severity of neurological deficits and their improvement in CSCI in older patients.

## Methods

The Japan Association of Spine Surgeons with Ambition performed a multicenter retrospective cohort study on inpatients aged ≥ 65 with cervical spinal cord and spine injury at 33 medical centers between 2010 and 2020, with a minimum follow-up period of 3 months. The Institutional Review Board of the representative facility (No. 3352-1) and each center approved the study protocol. The current study is a report presentation collected from similar data as other studies^14,15^ and was conducted in compliance with the Declaration of Helsinki.

A total of 1512 patients with CSCI were included in this study. The variables included the age at injury, sex, height, weight, body mass index (BMI), pre-injury activities of daily living (ADL), the mechanism of injury, the number of diagnosed with OPLL before the injury, the number of vertebral levels of OPLL, the level of signal intensity change on magnetic resonance imaging (MRI), the presence of bone injury, the presence of diffuse idiopathic skeletal hyperostosis, American Spine Injury Association (ASIA) impairment scale (AIS) grade at the injury, complication injuries during injury, comorbidity before the injury, treatment, the period before surgery, surgical approach, and perioperative complications. OPLL was found in 332 of all patients. Moreover, the overall proportion of OPLL was 22.0% of the CSCI cases in older adults.

### Analysis 1: OPLL vs. non-OPLL

We divided 1512 patients into two groups: those diagnosed with OPLL (OPLL group) and those without OPLL (non-OPLL group). The variables included the age at injury, sex, height, weight, BMI, smoking history, pre-injury ADL (independent walker or not), the presence of diabetes mellitus, dementia, cervical bone injury, signal intensity change on MRI, and surgical treatment. Injury mechanisms were classified as falling from the level ground (low energy) and more, such as high falls, traffic accidents, and others, including unspecified (high energy).

The moderator variables influencing the neurological prognosis (age, sex, BMI, pre-injury ADL, diabetes mellitus, dementia, bone injury, signal intensity change on MRI, and surgical treatment) were matched between the groups using propensity score matching (PSM). At baseline, 6 months, and 12 months of follow-up, the primary outcome measure comprised the ordinal change in the AIS grade and ASIA motor score (AMS). The time of admission was designated as baseline. We each assigned 5 points using 10 pairs of key muscles to evaluate the AMS. The scores ranged from 0 to 100. Higher scores in this range indicated stronger motor recovery. The secondary outcomes were morbidity and mortality from the baseline to 6 months and 12 months following injury.

### Analysis 2: OPLL vs. non-OPLL in CSCI without bone injury

In patients with CSCI without bone injury, we compared the OPLL and non-OPLL groups. The moderator variables included age, sex, BMI, pre-injury ADL, diabetes mellitus, dementia, signal intensity change on MRI, and surgical treatment. Similar to Analysis 1, we compared the primary and secondary outcomes by adjusting the PSM.

### Statistical analyses

Descriptive statistics for qualitative data are expressed as numbers and percentages, while quantitative data are expressed as the mean and standard deviation. We performed the Chi-square or Fisher’s exact tests and the t-test for the categorical and continuous variables, respectively. Following PSM, we conducted the McNemar test and paired t-test for the categorical and continuous variables, respectively.

Statistical test results were considered significant for p-values < 0.05, and all p-values were two-sided. All statistical analyses were performed with EZR (Saitama Medical Center, Jichi Medical University, Saitama, Japan), a graphical user interface for R (The R Foundation for Statistical Computing, Vienna, Austria, http://www.R-project.org/, version 4.1.1)^16^. More precisely, it is a modified version of the R commander designed to add frequently used statistical functions in biostatistics.

### Ethical approval

The institutional review board of representative facility reviewed and approved this study (Kanazawa University, No. 3352 1).

### Informed consent

Informed consent was obtained from all participants in this study.

## Results

**Figure 1:**
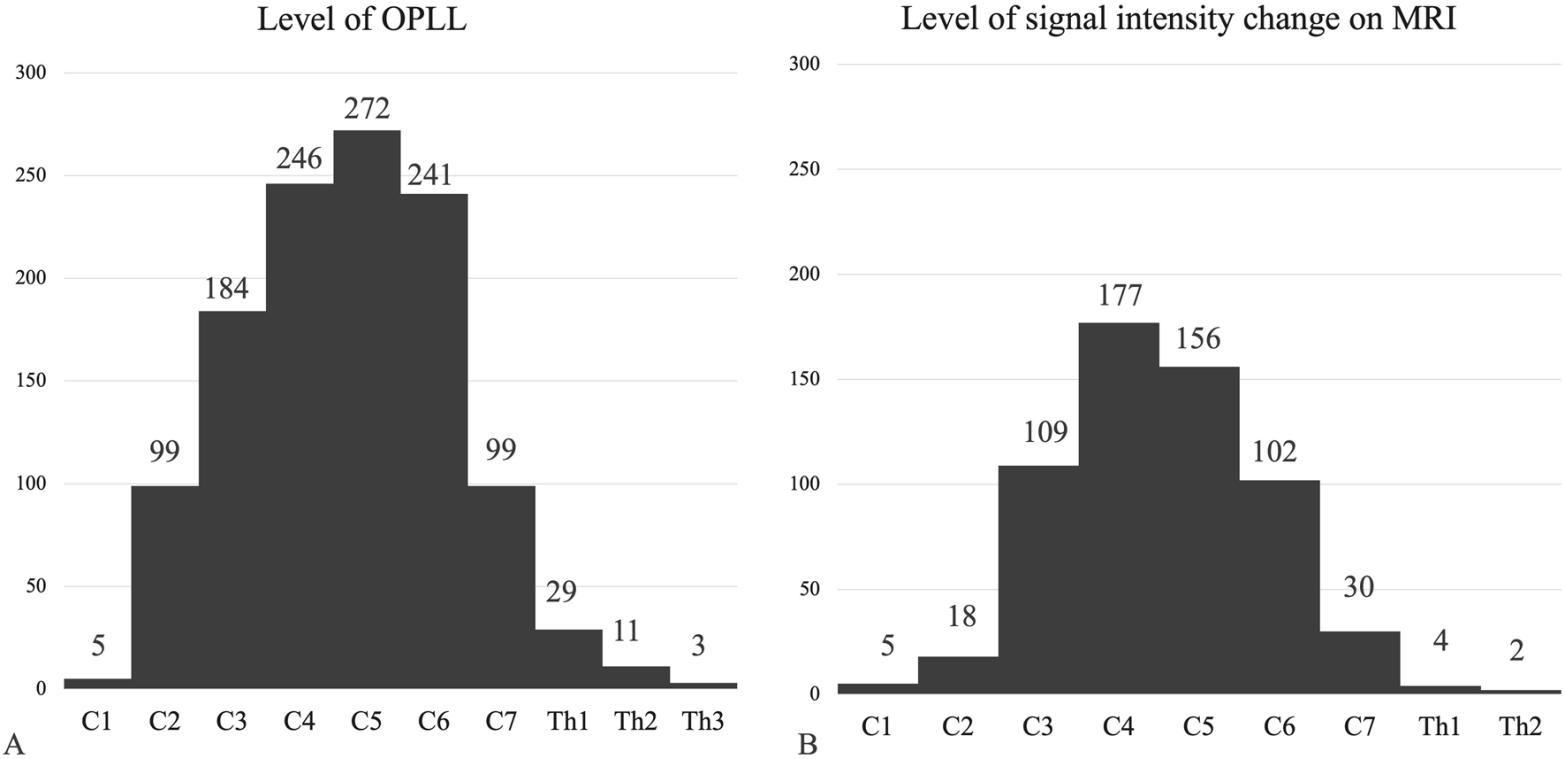
OPLL levels (**A**) and signal intensity changes on MRI (**B**). OPLL, ossification of the posterior longitudinal ligament; MRI, magnetic resonance imaging.

In the patients with OPLL in CSCI, the mean age was 75.3 ± 6.7 years, and the male-to-female ratio was 268:64, with men accounting for 80.7% of the population. The mean height and weight were 161.8 ± 8.7 cm and 59.6 ± 10.9 kg, respectively. Before the injury, 88.0% of patients could walk independently, and approximately half of the injuries were caused by ground-level falls. Sixty-four percent of patients had CSCI without bone injury, and 31.6% developed diffuse idiopathic skeletal hyperostosis. The AIS grade at the time of injury ranged from A to C in 46.4% of patients. Comorbidities before injury included hypertension in 50.9% of patients and diabetes mellitus in 26.2% of patients. Surgical treatment was performed in 67.7% of patients, and the mean waiting period for surgery was 21.6 ± 46.1 days. The posterior surgical approach was adopted in 97.8% of cases (Table 1). Figure 1 depicts the sum of the levels with OPLL and signal intensity changes detected on MRI. Of the 175 patients for whom conservative treatment was selected as the initial treatment, 68 (38.9%) were eventually converted to surgery. As for reasons for conversion to surgery, 46 cases were because of worsening or persistent symptoms, 6 were because of complications that initially made surgery not an option, and 16 cases were unknown.

**Table 1:** Demographic characteristics of older patients with CSCI with OPLL.

N = 332	Value
Age (yrs.)	75.3 ± 6.7
Sex (men:women)	268:64
Height (cm)	161.8 ± 8.7
Weight (kg)	59.6 ± 10.9
BMI (m/kg^2^)	22.7 ± 3.9
Pre-injury ADL (%)	
Walking independently	292 (88.0)
Walking with a cane	23 (6.9)
Walking with a walker	11 (3.3)
Others	6 (1.8)
Injury mechanism (%)	
Falling from the level ground	165 (49.7)
Falling from below one meter	43 (13.0)
Falling above one meter	62 (18.7)
Traffic accidents	45 (13.6)
Others	17 (5.1)
Diagnosed for OPLL before injury (%)	16 (4.8)
Cervical bone injuries (%)	
With	121 (36.4)
Without	211 (63.6)
With DISH (%)	105 (31.6)
AIS at injury (%)	
A	39 (11.7)
B	22 (6.6)
C	93 (28.0)
D	139 (41.9)
Without neurological disorder	37 (11.1)
Unknown	2 (0.6)
Comorbidity before the injury (%)	
Hypertension	169 (50.9)
Diabetes mellitus	87 (26.2)
Cardiovascular disease	55 (16.6)
Cerebrovascular disease	34 (10.2)
Malignant tumor	33 (9.9)
Renal disease	17 (5.1)
Osteoporosis	16 (4.8)
Dementia	16 (4.8)
Respiratory disease	11 (3.3)
Rheumatoid arthritis	8 (2.4)
Parkinson’s disease	5 (1.5)
Surgical treatment (%)	225 (67.8)
Time to surgery (days)	21.6 ± 46.1
Surgical approach (%)	
Posterior	220 (97.8)
Anterior	4 (1.7)
Anterior and posterior	1 (0.3)

CSCI, cervical spine cord injury; OPLL, ossification of the posterior longitudinal ligament; AIS, American Spine Injury Association impairment scale; DISH, diffuse idiopathic skeletal hyperostosis; and ADL, activities of daily living.

Table 2 summarizes surgery-related and in-hospital complications.

**Table 2:** Surgery-related and in-hospital complications.

Surgery-related complications (%)	N = 225	In-hospital complications (%)	N = 332
Dural tear	4 (1.8)	Death	19 (5.7)
Extubation difficulties	2 (0.9)	Pneumonia	8
Spinal cord injury	1 (0.4)	Airway obstruction	3
Massive bleeding	2 (0.9)	Intestinal necrosis	1
Radial nerve palsy	1 (0.4)	Massive bleeding	1
SSI	6 (2.7)	Unknown	6
C5 palsy	4 (1.8)	Pneumonia	45 (13.6)
Worsening radiculopathy	2 (0.9)	Urinary tract infection	38 (11.4)
Epidural hematoma	1 (0.4)	Respiratory failure	36 (10.8)
Implant failure	1 (0.4)	Dysphagia	34 (10.2)
Reoperation	9 (4.0)	Delirium	26 (7.8)
Infection	3	Thromboembolism	8 (2.4)
Additional decompression	2	Cerebral infarction	3 (0.9)
Additional fixation	1	Retropharyngeal hematoma	1 (0.3)
Screw replacement	1	Others	15 (4.5)
Dysphagia	2		

SSI, surgical site infection.

### Analysis 1: OPLL vs. non-OPLL

There were 332 patients in the OPLL group and 1,180 in the non-OPLL group. Patients in the OPLL group displayed a higher men-to-women ratio (80.7% vs. 62.6%, p < 0.001), higher BMI (22.7 ± 3.9 vs. 21.7 ± 4.2, p < 0.001), higher rate of smoking history (39.8% vs. 27.1%, p = 0.001), higher prevalence of low energy trauma (50.2% vs. 35.2%, p < 0.001), higher prevalence of diabetes mellitus (26.7% vs. 21.0%, p = 0.035), a higher proportion of signal intensity change on MRI (78.1% vs. 57.9%, p < 0.001), and a higher proportion of surgical treatment (67.8% vs. 57.5%, p = 0.001) than the non-OPLL group (Table 3). The baseline AIS grade was not significantly different between the groups before matching (p = 0.630). In contrast, the baseline AMS in the OPLL group was significantly lower than in the non-OPLL group (55.6 ± 34.2 vs. 60.7 ± 32.7, p = 0.029). Patients in the OPLL group displayed a higher in-hospital complication rate than those in the non-OPLL group (38.0% vs. 30.6%, p = 0.013). There were no significant differences in the in-hospital mortality between the groups (5.8% vs. 3.9%, p = 0.170) (Table 4). Following PSM of the baseline characteristics, both groups had 279 patients. The AIS grade and AMS from baseline were not significantly different between each group. There were no significant differences in the in-hospital complication rate. However, the OPLL group displayed significantly higher in-hospital mortality than the non-OPLL group (5.4% vs. 1.4%, p = 0.010). Changes in the AIS grade and AMS from the baseline to 6 months and 12 months following injury were not significantly different between the groups. There was no significant difference in the comorbidity and mortality at 6 months and 12 months following injury (Table 4).

**Table 3:** A comparison of the demographic data at baseline.

	Before PSM			After PSM		
	OPLL (N = 332)	Non-OPLL (N = 1180)	p-value	OPLL (N = 279)	Non-OPLL (N = 279)	p-value
Age (yrs.)	75.3 ± 6.7	76.0 ± 7.0	0.099	74.9 ± 6.4	74.7 ± 6.2	0.72
Sex (%)						
Men	268 (80.7)	739 (62.6)	< 0.001	224 (80.3)	223 (79.9)	1
Women	64 (19.3)	441 (37.4)		55 (19.7)	56 (20.1)	
BMI (m/kg^2^)	22.7 ± 3.9	21.7 ± 4.2	< 0.001	22.5 ± 3.8	22.8 ± 3.4	0.39
Smoking history (%)	86 (39.8)	202 (27.1)	0.001	72 (39.6)	76 (43.7)	0.45
Diabetes mellitus (%)	87 (26.7)	243 (21.0)	0.035	73 (26.2)	73 (26.2)	1
Dementia (%)	13 (4.0)	82 (7.1)	0.054	8 (2.9)	8 (2.9)	1
Independent walker (%)	292 (89.0)	1049 (89.7)	0.76	253 (90.7)	258 (92.5)	0.54
Low energy trauma	165 (50.2)	414 (35.2)	< 0.001	138 (49.5)	133 (47.7)	0.74
Without cervical fracture (%)	211 (63.7)	403 (34.2)	< 0.001	177 (63.4)	161 (57.7)	0.19
Signal intensity change on MRI (%)	246 (78.1)	617 (57.9)	< 0.001	217 (77.8)	227 (81.4)	0.35
Surgical treatment (%)	225 (67.8)	678 (57.5)	0.001	190 (68.1)	207 (74.2)	0.14

PSM, propensity score matching; BMI, body mass index; MRI, magnetic resonance imaging.

**Table 4:** A comparison of the primary and secondary outcomes between the OPLL and non-OPLL groups.

	Before PSM			After PSM		
	OPLL (N = 332)	Non-OPLL (N = 1180)	p-value	OPLL (N = 279)	Non-OPLL (N = 279)	p-value
Baseline						
AIS (%)						
A	39 (13.3)	86 (11.4)	0.63	33 (13.3)	20 (8.5)	0.40
B	22 (7.5)	46 (6.1)		18 (7.2)	20 (8.5)	
C	93 (31.7)	243 (32.2)		81 (32.5)	78 (33.2)	
D	139 (47.4)	379 (50.3)		117 (47.0)	117 (49.8)	
AMS	55.6 ± 34.2	60.7 ± 32.7	0.029	56.3 ± 33.7	57.3 ± 31.8	0.73
Complication (%)	124 (38.0)	359 (30.6)	0.013	107 (38.6)	90 (32.3)	0.13
Death (%)	19 (5.8)	46 (3.9)	0.17	15 (5.4)	4 (1.4)	0.010
6 months						
AIS improvement (%)						
Worsening	2 (1.1)	3 (0.6)	0.62	2 (1.3)	1 (0.5)	0.58
No improvement	112 (59.9)	306 (56.1)		94 (59.1)	99 (53.5)	
Improvement	63 (33.7)	205 (37.6)		56 (35.2)	73 (39.5)	
At least a 2-grade improvement	10 (5.3)	31 (5.7)		7 (4.4)	12 (6.5)	
AMS improvement	15.6 ± 19.3	15.4 ± 19.3	0.87	16.2 ± 19.9	17.8 ± 20.0	0.48
Comorbidity (%)	22 (9.6)	63 (7.2)	0.21	16 (8.5)	17 (7.4)	0.72
Mortality (%)	1 (0.4)	0 (0.0)	0.21	1 (0.5)	0 (0.0)	0.45
12 months						
AIS improvement (%)						
Worsening	1 (0.8)	0 (0.0)	0.41	1 (0.9)	0 (0.0)	0.50
No improvement	71 (54.6)	210 (52.4)		61 (54.0)	67 (50.4)	
Improvement	50 (38.5)	161 (40.1)		45 (39.8)	54 (40.6)	
At least a 2-grade improvement	8 (6.2)	30 (7.5)		6 (5.3)	12 (9.0)	
AMS improvement	17.7 ± 22.9	16.8 ± 20.5	0.66	18.5 ± 23.6	20.0 ± 21.7	0.62
Comorbidity (%)	15 (9.3)	32 (5.0)	0.058	12 (8.8)	7 (4.3)	0.15
Mortality (%)	1 (0.6)	4 (0.6)	1	1 (0.7)	3 (1.9)	0.63

PSM, propensity score matching; OPLL, ossification of the posterior longitudinal ligament; AIS, American Spine Injury Association impairment scale; and AMS, American Spine Injury Association motor score.

### Analysis 2: OPLL vs. non-OPLL in CSCI without bone injury

There were 221 patients with CSCI without bone injury in the OPLL group and 403 in the non-OPLL group. Patients in the OPLL group demonstrated younger age (74.4 ± 6.6 vs. 75.9 ± 6.7, p = 0.008), higher men-to-women ratio (78.7% vs. 68.2%, p = 0.006), higher BMI (22.8 ± 4.2 vs. 22.0 ± 4.0, p = 0.020), higher rate of smoking history (39.8% vs. 27.1%, p = 0.001), and a higher proportion of surgical treatment (61.6% vs. 44.2%, p < 0.001) than those in the non-OPLL group^14^ (Table5). Patients in the OPLL group displayed a lower baseline AIS grade (p = 0.032) and baseline AMS (58.2 ± 32.8 vs. 65.2 ± 29.2, p = 0.009) than those in the non-OPLL group before matching^15^. There were no significant differences in the in-hospital complications and mortality between the groups (28.0% vs. 22.9%, p = 0.166; 2.4% vs. 2.0%, p = 0.770) (Table 6). Following PSM of the baseline characteristics, both groups had 176 patients. The AIS grade from baseline was significantly lower in the OPLL group than in the non-OPLL group (p = 0.045). Changes in the AIS grade and AMS from the baseline to 6 months and 12 months post-injury were not significantly different between the groups. There were only significant differences in the comorbidity at 12 months following injury (8.2% vs. 1.0%, p = 0.016) (Table 6).

**Table 5:** Patient demographics in cervical spinal cord injury without bone injury.

	Before PSM			After PSM		
	OPLL (N = 211)	Non-OPLL (N = 403)	p-value	OPLL (N = 176)	Non-OPLL (N = 176)	p-value
Age (yrs.)	74.4 ± 6.6	75.9 ± 6.7	0.008	74.3 ± 6.1	74.8 ± 6.7	0.46
Sex (%)						
Men	166 (78.7)	275 (68.2)	0.006	136 (77.3)	129 (73.3)	0.46
Women	45 (21.3)	128 (31.8)		40 (22.7)	47 (26.7)	
BMI (m/kg^2^)	22.8 ± 4.2	22.0 ± 4.0	0.020	22.6 ± 4.2	22.4 ± 3.7	0.58
Smoking history (%)	61 (44.9)	75 (30.4)	0.005	53 (45.7)	42 (36.8)	0.18
Diabetes mellitus (%)	60 (29.0)	112 (28.4)	0.92	52 (29.5)	49 (27.8)	0.81
Dementia (%)	6 (2.9)	22 (5.6)	0.16	4 (2.3)	6 (3.4)	0.75
Independent walker (%)	188 (90.4)	352 (88.2)	0.50	161 (91.5)	161 (91.5)	1
Low energy trauma	114 (54.8)	211 (52.5)	0.61	93 (52.8)	94 (53.4)	1
Signal intensity change on MRI (%)	179 (87.3)	327 (82.6)	0.16	152 (86.4)	160 (90.9)	0.24
Surgical treatment (%)	130 (61.6)	178 (44.2)	< 0.001	109 (61.9)	122 (69.3)	0.18

PSM, propensity score matching; BMI, body mass index; MRI, magnetic resonance imaging; and OPLL, ossification of the posterior longitudinal ligament.

**Table 6:** A comparison of the primary and secondary outcomes between the OPLL and non-OPLL groups with CSCI without bone injury.

	Before PSM			After PSM		
	OPLL (N = 211)	Non-OPLL (N = 403)	p-value	OPLL (N = 176)	Non-OPLL (N = 176)	p-value
Baseline						
AIS (%)						
A	18 (8.6)	16 (4.0)	0.032	16 (9.1)	4 (2.3)	0.045
B	15 (7.1)	16 (4.0)		11 (6.3)	11 (6.3)	
C	71 (33.8)	144 (35.8)		60 (34.3)	69 (39.4)	
D	106 (50.5)	226 (56.2)		88 (50.3)	91 (52.0)	
AMS	58.2 ± 32.8	65.2 ± 29.2	0.009	58.6 ± 32.3	61.7 ± 29.1	0.36
Complication (%)	58 (28.0)	92 (22.9)	0.17	49 (28.0)	37 (21.0)	0.14
Death (%)	5 (2.4)	8 (2.0)	0.77	3 (1.7)	3 (1.7)	1
6 months						
AIS improvement (%)						
Worsening	1 (0.7)	2 (0.7)	0.19	1 (0.9)	1 (0.7)	0.12
No improvement	87 (62.1)	169 (56.9)		72 (61.5)	69 (49.6)	
Improvement	43 (30.7)	116 (39.1)		37 (31.6)	63 (45.3)	
At least a 2-grade improvement	9 (6.4)	10 (3.4)		7 (6.0)	6 (4.3)	
AMS improvement	16.5 ± 19.9	16.1 ± 18.1	0.85	17.3 ± 20.6	17.8 ± 19.2	0.83
Comorbidity (%)	12 (8.0)	23 (7.7)	1	8 (6.4)	5 (3.4)	0.27
Mortality (%)	0	0	NA	0	0	NA
12 months						
AIS improvement (%)						
Worsening	0	0	0.45	0	0	0.26
No improvement	58 (58.6)	113 (53.6)		53 (60.2)	48 (48.5)	
Improvement	34 (34.3)	87 (41.2)		29 (33.0)	44 (44.4)	
At least a 2-grade improvement	7 (7.1)	11 (5.2)		6 (6.8)	7 (7.1)	
AMS improvement	18.5 ± 24.1	18.1 ± 19.5	0.88	19.2 ± 24.7	19.6 ± 20.7	0.90
Comorbidity (%)	10 (8.9)	6 (2.8)	0.027	8 (8.2)	1 (1.0)	0.016
Mortality (%)	1 (0.9)	1 (0.5)	1	1 (1.0)	0 (0.0)	0.49

PSM, propensity score matching; OPLL, ossification of the posterior longitudinal ligament; CSCI, cervical spinal cord injury; AIS, American Spine Injury Association impairment scale; and AMS, American Spine Injury Association motor score.

## Discussion

This large multicenter study investigated the epidemiology of CSCI with OPLL in older adults. Our results showed that CSCI occurred in concomitance with OPLL in 22.0% of the older population. The male-to-female ratio in the OPLL group was approximately 4:1. Half of all patients experienced low-energy trauma-induced injury, and one-third had CSCI without bony injury. A total of 279 pairs were created using PSM. There was no significant difference in the AIS grade and AMS between patients with and without OPLL during hospitalization, and 6 and 12 months after injury. Our findings suggested that patients with CSCI with OPLL can be expected to improve in a manner similar to that in patients without OPLL.

The prevalence of OPLL within a Japanese older patient population with CSCI was 22.0% and that of CSCI without bone injury in older adults was 34.4%. Kawano et al.^17^ reported that 22.2% of patients with traumatic CSCI had OPLL, and Boody et al.^18^ reported that approximately 30% of the patients with CSCI had OPLL. Endo et al.^19^ identified OPLL in 6.5% of the patients with CSCI. In contrast, Okada et al.^20^ reported OPLL in 10.1% of the patients with CSCI. Chikuda et al.^4^ reported that 34% of those with CSCI without bone injury had OPLL, compared with 38% of patients identified by Koyanagi et al.^21^. Approximately 26–38% of CSCI cases without bone injury are associated with OPLL^22,23^. Our results are similar to previous reports.

Regarding sex differences in OPLL, the men-to-women ratio was 80.7% in the older adults with CSCI and OPLL. Ohtsuka et al.^24^ reported that the prevalence of OPLL was 4.3% and 2.4% in men and women, respectively, in an X-ray survey of healthy Japanese. Previous observational studies demonstrated that the prevalence of OPLL in the general population is approximately two-fold higher in men than in women^25,26^. A nationwide survey in Japan showed that the men-to-women ratio was 3:1 in those with traumatic CSCI^2^. Our study displayed a high proportion of men, nearly four times that of women, considering the prevalence of OPLL in the general population. In older adults with OPLL, we observed a higher proportion of men with CSCI.

The OPLL group tended to display severe paralysis during the injury. After adjusting for the background variables affecting the neurological findings during injury, there were no significant differences in the AIS grade and AMS between the OPLL and non-OPLL groups, except for the AIS grade in patients with CSCI without bone injury. There was no significant difference in the rate of improvement in the neurological findings between patients with and without OPLL in either the AIS grade or AMS. Few reports have compared the rate of improvement of the neurological findings in patients with and without OPLL. These results indicated that the presence of OPLL exacerbates the neurological symptoms at the time of injury, but it had less impact on the recovery process of the neurological symptoms.

In this study, the in-hospital complication rate was 9.6% and 7.2% in the OPLL and non-OPLL groups, respectively, which was not significantly different and lower than in previous reports. However, patients with CSCI are frail and have a significant risk of complications. In previous reports, the in-hospital complication rate for spine surgery in older adults was approximately 20%, with hemorrhage, delirium, and UTI as the most common complications^27,28^. Bernstein et al.^29^ reported that the number of surgical cases of OPLL has increased significantly and provided national estimates for 21% of inpatient postoperative complications. For patients requiring surgical treatment for degenerative cervical myelopathy, OPLL can present a significant surgical challenge, with complication rates ranging from 5.2 to 57.6%^30^. Moreover, in a prospective, multicenter study, OPLL was an independent risk factor for perioperative complications in patients surgically treated for cervical myelopathy^31^. One factor contributing to the low complication rate in this study was that morbidity in retrospective studies is not calculated as accurately as in prospective studies and may be underestimated^32^. The comorbidity rate at 6 months and 12 months following injury was higher in the OPLL group with CSCI without bone injury, even after adjusting for the PSM using background variables. There was no significant difference in mortality between the groups. Similar to previous reports, we observed a trend towards a higher complication rate in patients with OPLL, and the OPLL group demonstrated a tendency to have greater complications.

This study has several limitations. First, it was not excluded from sampling bias because it cannot be extracted from medical records. Second, we did not evaluate the ossification type. There was no information available about the length and thickness of OPLL, which might be correlated with CSCI. We did not comprehensively investigate the relationship between the diameter of the spinal canal, the degree of cord compression, and the risk of myelopathy. Therefore, this necessitated further research on the morphology of OPLL. Third, the treatment contents and policies are not standardized among facilities, a limitation in a retrospective multicenter study; thus, prospective studies are desirable in the future. Fourth, the 12-month follow-up after PSM may have insufficient power to compare the mortality and morbidity in a reduced sample size. However, this novel study analyzed a large amount of sample data comparison among patients with or without OPLL. Prospective studies are preferred for an accurate assessment of morbidity and mortality.

## Conclusions

The prevalence of OPLL in CSCI was 22.0% in older adults. Patients with OPLL had a higher proportion of men, higher BMI, higher smoking history rate, greater injuries owing to low energy as falling from the level ground, higher prevalence of diabetes mellitus, a higher proportion of signal intensity changes on MRI, and a higher proportion of surgical treatment.

In this study, patients with OPLL tended to display worse neurological findings during the injury; nonetheless, OPLL was not associated with poorer neurological improvement after CSCI.

## Data Availability

The study data and materials’ details may be made available upon reasonable request by e-mail the corresponding author.
